# Development of a Patient-Reported Outcomes Tool to Assess Pain and Discomfort in Autosomal Dominant Polycystic Kidney Disease

**DOI:** 10.2215/CJN.0000000000000034

**Published:** 2023-01-02

**Authors:** Dorothee Oberdhan, Jason C. Cole, Mark J. Atkinson, Holly B. Krasa, Sara N. Davison, Ronald D. Perrone

**Affiliations:** 1 Otsuka Pharmaceutical Development & Commercialization, Inc., Rockville, Maryland; 2 P3 Research Consulting, LLC, Torrance, California; 3 COA Evidentiary Analytics, LLC, Powers, Oregon; 4 Department of Family Medicine and Public Health, University of California San Diego, San Diego, California; 5 Blue Persimmon Group, LLC, Washington, DC; 6 Department of Medicine, University of Alberta, Edmonton, Canada; 7 Division of Nephrology, Tufts Medical Center, Tufts University School of Medicine, Boston, Massachusetts

**Keywords:** ADPKD, chronic kidney disease, genetic renal disease, polycystic kidney disease, quality of life, patient reported outcome measures, pain

## Abstract

**Background:**

Pain has been identified as a core outcome for individuals with autosomal dominant polycystic kidney disease (ADPKD), but no disease-specific pain assessment has been developed using current development methodology for patient-reported outcomes (PRO) instruments. We developed and validated an ADPKD-specific pain questionnaire: the ADPKD Pain and Discomfort Scale (ADPKD-PDS).

**Methods:**

Conceptual underpinnings were drawn from literature review, concept elicitation, expert consultation, and measurement performance. In the qualitative analysis phase, concepts were elicited from focus groups of adults with ADPKD, and the resulting draft instrument was refined using cognitive debriefing interviews with individuals with ADPKD. For quantitative analysis, adults with ADPKD completed the draft instrument and other PRO tools in an online survey, and a follow-up survey was conducted 3–4 weeks later. Survey responses were analyzed for item-level descriptive statistics, latent model fit statistics, item discrimination, item- and domain-level psychometric statistics, test-retest reliability, responsiveness to change, and convergent validity.

**Results:**

In the qualitative phase, 46 focus groups were conducted in 18 countries with 293 participants. Focus groups described three conceptually distinct types of ADPKD-related pain and discomfort (dull kidney pain, sharp kidney pain, and fullness/discomfort). In the quantitative phase, 298 adults with ADPKD completed the online survey, and 108 participants completed the follow-up survey. After iterative refinement of the instrument, latent variable measurement models showed very good fit (comparative fit and nonnormed fit indices both 0.99), as did item- and domain-level psychometric characteristics. The final ADPKD-PDS contains 20 items assessing pain severity and interference with activities over a 7-day recall period.

**Conclusions:**

The ADPKD-PDS is the first validated tool for systematically assessing pain and discomfort in ADPKD.

## Visual Abstract

**Figure:**
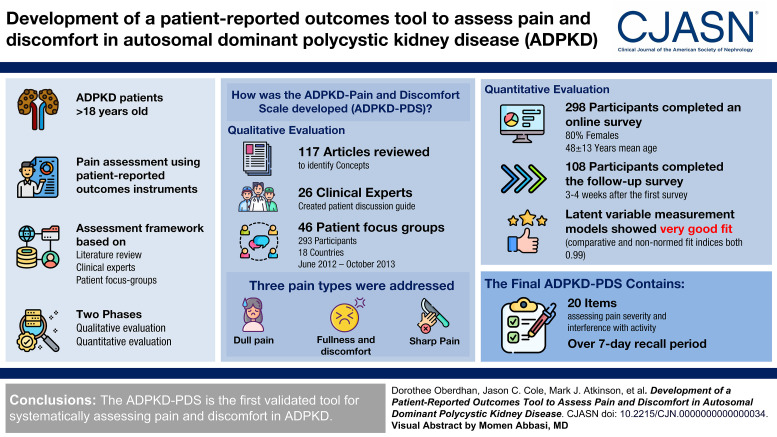

## Introduction

Autosomal dominant polycystic kidney disease (ADPKD) is a progressive systemic disease characterized by kidney enlargement and reduced kidney function.^1–3^ Although many individuals with ADPKD appear asymptomatic, approximately 60% experience acute and/or chronic abdominal, back, or flank pain across the disease spectrum regardless of age, and pain is the most frequent symptom leading to diagnosis.^2–5^

To assess health-related quality of life from the patient's perspective, patient-reported outcomes (PRO) instruments are used as outcome measures in clinical trials, and in practice settings, to guide treatment decisions.^6^ Pain is most appropriately self-reported by patients to obtain direct assessment of pain severity and the patient's perspective on pain impacts.^7^ Clinical trials in ADPKD typically report outcomes such as kidney volume and function but infrequently provide data on PROs, despite the significant impact of symptoms, emotional distress, and ability to perform activities of daily living. To address this unmet need, the Standardized Outcomes in Nephrology-Polycystic Kidney Disease (SONG-PKD) initiative obtained input from individuals with ADPKD, caregivers, and health care providers to identify core outcomes.^8^ People with ADPKD and clinicians both consider pain the most important patient-centric outcome,^9^ although patients may under-report chronic pain to their health care providers.^8^ The 2021 Polycystic Kidney Disease Regulatory Summit identified pain as a core outcome for ADPKD research.^10,11^ Yet unclear conceptualization of pain has so far led to its inconsistent measurement across studies.^12^

The objective of this research was to develop a PRO instrument to assess ADPKD-associated pain and discomfort through the establishment of a meaningful conceptual framework and robust content and construct validation.

## Methods

**Figure 1:**
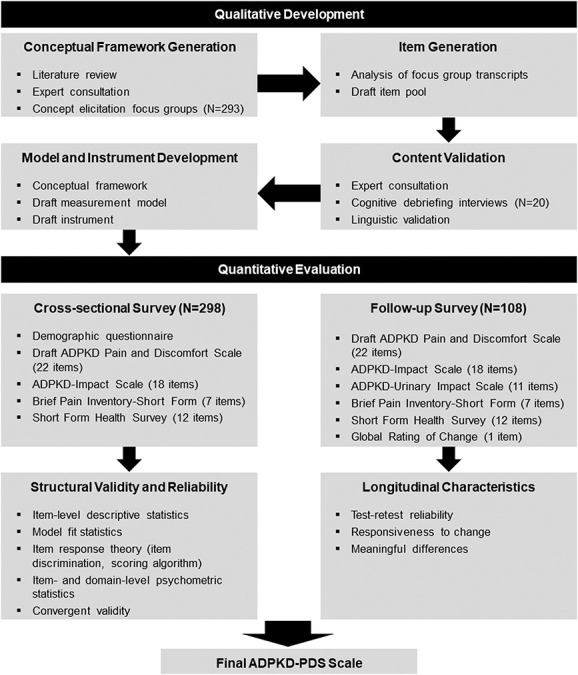
**Qualitative and quantitative steps in the development of the ADPKD-PDS.** ADPKD, autosomal dominant polycystic kidney disease; ADPKD PDS, ADPKD Pain and Discomfort Scale.

Development of the ADPKD Pain and Discomfort Scale (ADPKD-PDS) followed standard methods for PRO instrument development (Figure 1).^13–15^ The New England Institutional Review Board (Needham, MA) served as the central review body for all components of this research. Participants were men and women aged ≥18 years with ADPKD recruited *via* physician and advocacy group referrals or print advertising who provided informed consent before study activities.

### Development of an ADPKD-Related Pain Framework and Questionnaire

We conducted a literature search, as described in the Supplemental Material, for disease-related pain symptoms, burden, impacts, and PROs to develop an initial assessment framework for pain and discomfort in ADPKD. Clinical experts provided their perspectives during advisory meetings. Concepts from the literature review and clinician discussions informed creation of a patient discussion guide for semistructured concept elicitation focus groups.

Focus groups were conducted in 18 countries to understand ADPKD symptoms and the intensity, burden, and impact of pain on health-related quality of life from the patient perspective (Supplemental Material). Discussion transcripts were deidentified to protect participant privacy and coded for concepts or themes. Concepts were identified on the basis of themes that were mentioned by ≥2 participants. Saturation was achieved when no new concepts or themes were identified during subsequent focus groups.

On the basis of focus group input, the initial framework was updated, and an item pool was generated for review with two PRO measurement experts and several clinical experts. The resulting initial version of the ADPKD-PDS was reviewed for translatability issues by linguistic specialists and reviewed with patients in the United States for clarity and understandability of content, instructions, items, and response options. Three rounds of cognitive debriefing interviews were performed, with each round testing iterative refinements that were summarized in an item-tracking matrix.

### Quantitative Evaluation

The updated ADPKD-PDS was tested in a quantitative study using an online survey format hosted by a third-party internet hosting firm (Toluna) to establish its measurement properties. Eligible participants were male or female, ≥18 years old, diagnosed with ADPKD, and had information available for CKD stage and total kidney volume from the previous 12 months. The requirement for total kidney volume was later relaxed after it was found that it was not readily available in the majority of the target population. Participants with a history of kidney transplantation were excluded. With a target enrollment of 300 participants, the PKD Foundation (Kansas City, MO) sent an email invitation to 2357 members who had opted to receive such information. Respondents were sent a web link to a consent form and screening questionnaire. Eligible participants proceeded to the baseline survey, which included a demographic questionnaire, ADPKD-PDS, ADPKD Impact Scale (ADPKD-IS), Brief Pain Inventory Short Form (BPI-SF), and 12-Item Short-Form Health Survey version 2 (SF-12v2).^16–18^

Completers of the initial survey were invited to participate in a follow-up survey, with invitations sent 25 days after a participant completed the first survey. Enrollment for this follow-up survey was performed on a rolling basis until the recruitment target of 100 was met. The follow-up survey was conducted using the same questionnaires administered at baseline plus a pain-based global rating of change.^19^ Because data were captured *via* an internet survey and were less vulnerable to data collection irregularities than in longitudinal clinical studies, no missing response values were expected or observed from participants of this online survey series.

### Analyses

In an initial data quality analysis, responses to individual items were evaluated for completeness, response frequency distributions, sufficient response variability, and symmetry of response distributions. We then examined structural validity, the degree to which the scores of an instrument adequately represent the overall construct it purports to measure,^20^ using confirmatory factor analysis and item response theory. Confirmatory factor analysis was used to determine whether the proposed organization of items by domains in the initial conceptual framework was consistent with the empirically observed data and, if necessary, to adjust the underlying measurement model. The item response theory analysis was conducted to ensure that item response categories were correctly ordered, covered the range of the dimensions (*e.g.*, pain severity or pain interference) being measured, and specified the most appropriate scoring method. Differential item function analyses evaluated whether item responses were affected by respondent sex.^21^ Additional analyses evaluated the adequacy of the correlations between item and domain scores and the internal consistency of the domains using standard psychometric methods and criteria.

Convergent validity^22^ of the ADPKD-PDS was assessed using other PRO measures of related constructs (BPI-SF, SF-12v2, ADPKD-IS). ADPKD-PDS Pain Severity was expected to be highly related to BPI-SF Intensity, and the ADPKD-PDS pain interference should be highly related to BPI-SF Impact and the SF-12v2 Physical Component Summary and Mental Component Summary. Both ADPKD-PDS scales were expected to have medium correlations with the ADPKD-IS scales. The test-retest reliability of the ADPKD-PDS was examined using test-retest intraclass correlation coefficients. Finally, to determine whether ADPKD-PDS domain scores could identify meaningful change over time, we divided participants into three responder categories (improvement in pain/discomfort, worsening of pain/discomfort, and no change) and conducted an anchor-based analysis using analysis of variance with the BPI-SF and pain-based global rating of change as the references for change. Analyses were conducted using Statistical Analysis Software (SAS) v9.1.3 (SAS Institute Inc., Cary, NC); STATA 14.1 (STATA Corp., College Station, TX); Mplus, v7.4 (Muthén & Muthén, Los Angeles, CA)^23^; and Xcalibre, v4 (Assessment Systems, Minneapolis, MN).

## Results

### Qualitative Development

The literature review identified 117 articles. ADPKD-related pain was most commonly described by pain location (low back, abdominal, head, chest, leg), intensity, and frequency. Few dedicated PRO measures include the concept of pain in ADPKD. Two exceptions are the ADPKD-IS^16^ and the Polycystic Liver Disease questionnaire,^24^ which are both validated for use in ADPKD and include pain assessments. These tools, however, do not provide in-depth evaluation of specific types and impacts of pain in ADPKD. One measure of ADPKD-related pain was developed by Bajwa *et al.*, but it was not on the basis of patient-centered concept elicitation that allows patient perspectives to shape the content and terminology used.^4,13^ Another, more recent, effort to standardize measurement of ADPKD-related pain is an assessment tool described by El-Damanawi *et al.* that utilizes domains of existing non-ADPKD-specific PROs on the basis of patient input.^25^

Concepts identified from the literature were discussed with 26 clinical experts from North America and Europe, with pain mostly considered a severe acute problem resulting from cyst ruptures or infections, or a milder chronic problem caused by posture-related issues due to kidney size or internal pressure from growing kidneys and described as discomfort.

**Figure 2:**
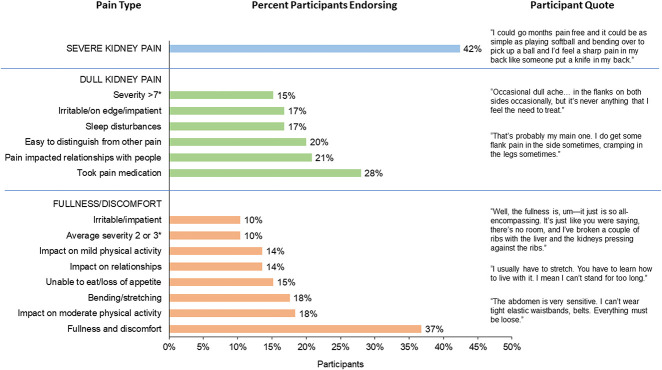
**Percentage of participants endorsing pain concepts during initial focus groups (*N*=125) with sample quotes**. *On a scale of 1–10.

A total of 293 patients across 18 countries participated in 46 focus groups from June 2012 through October 2013 (Table 1) to account for expected cultural influences on pain reporting and pain experience. Discussions during the initial focus groups (*n*=125) revealed that individuals with ADPKD experience three distinct types of pain: dull kidney pain, a sensation of fullness and discomfort, and sharp kidney pain, which is most often associated with an event such as rupture of a cyst or infection. The percentages of participants endorsing pain concepts in these initial discussions are presented with sample quotes in Figure 2. On the basis of patient input, the discussion guide was updated to address the three pain types and their impact. Participants in subsequent groups (*n*=168) readily endorsed the new pain categories, and concept elicitation quickly reached data saturation. No differences in the pain experience and effects were identified by country.

**Table 1:** Locations of autosomal dominant polycystic kidney disease focus groups and number and sex of participants

Country	Number of Focus Groups	Number of Participants		
		Male	Female	Total
**Initial focus groups**				
Germany	4^a^	11	19	30
Switzerland	2	7	9	16
Turkey	2	8	6	14
United Kingdom	2	5	7	12
United States	7	25^b^	28	53
**Follow-on focus groups**				
Argentina	2	5	4	9
Australia	1^a^	4	4	8
Brazil	2	4	5	9
Canada	2	2	6	8
China	2^a^	10	5	15
Czech Republic	2	7	8	15
Hungary	2	3	3	6
Japan	2^a^	3^b^	9^b^	12
Poland	2	4	4	8
Romania	2^a^	3^b^	4	7
South Korea	2	8	8	16
Spain	2^a^	8	8	16
Taiwan	2	8	8	16
United States	4	12	11	23
Total	46	137	156	293

a One group in Germany, the group in Australia, both groups in China, one group in Japan, one group in Romania, and both groups in Spain included males and females, as the groups were too small to split by sex. Participants were advised before the focus group that some sensitive issues might be discussed that could be embarrassing in front of the other sex, and participants were asked if they were still willing to participate. Participants were reminded of this issue before the groups began.

b Three males in the United States, two males and four females in Japan, and one male in Romania were unable to attend the focus group and were interviewed individually.

Expert review of the conceptual framework and draft questionnaire resulted in updates to improve the clarity of instructions, items, and response options.

Cognitive debriefing interviews were conducted with ten participants in round 1, five in round 2, and five in round 3, all from December 2013 through March 2015. On the basis of the interviews and linguistic review, we made additional minor wording and layout updates. The resultant draft ADPKD-PDS consisted of 22 items with a five-category rating scale (ranging from low/no symptoms or interference to severe symptoms or complete interference), with responses on the basis of a 1-week recall period.

### Measurement Properties

A total of 445 of 2357 invited individuals (19%) responded to the online survey invitation. Of these, 135 did not meet enrollment criteria, and 12 were excluded due to missing data, leaving 298 participants. Table 2 shows the participant characteristics of the baseline and follow-up surveys conducted in 2015.

**Table 2:** Survey study: demographic and clinical characteristics of participants

Characteristic	Baseline (*N*=298)	Follow-up (*N*=108)
**Age, yr**		
Mean (SD)	48 (13)	47 (13)
Median (range)	49 (20–90)	48 (20–75)
**Sex, *n* (%)**		
Male	59 (20)	20 (19)
Female	239 (80)	88 (82)
**Ethnicity, *n* (%)**		
Asian	7 (2)	0 (0)
Black	8 (3)	3 (3)
Hispanic/Latino	17 (6)	6 (6)
White	259 (87)	97 (90)
Other	7 (2)	2 (2)
**CKD stage, *n* (%)**		
1	79 (27)	35 (32)
2	61 (20)	18 (17)
3	88 (30)	31 (29)
4	33 (11)	11 (10)
5	28 (9)	10 (9)
Don't know	9 (3)	3 (3)
**Time since diagnosis, yr, *n* (%)**		
0–5	40 (13)	—
6–10	55 (19)	—
11–20	92 (31)	—
21–30	72 (24)	—
≥31	39 (13)	—
Receiving dialysis, *n* (%)	16 (5)	—
Mean age (range) at first dialysis	42 (6–69)	—

Percentages may not add up to 100 due to rounding.

Results for standard psychometric analyses are described in the Supplemental Material, including item response theory, differential item performance, item- and domain-level statistics, convergent validity, test-retest reliability, and responsiveness to change.

### Item-Level Descriptive Statistics

Distributions for all items were non-normal (Supplemental Table 2). Seventeen of 22 items had appreciable skew (exceeding a Z-score threshold of 3 SD units). However, kurtosis Z-scores indicated a shape closer to normal (these scores reflect height to length ratio, or peakedness, of the distribution curve and were between ±3 SD for 13 items). The percentage of participants reporting the lowest score for an item (floor effects) ranged from 22% to 70%. Participants reporting the highest item score (ceiling effects) exceeded 10% for only one item (Sharp Pain Severity at its worst).

### Confirmatory Factor Analysis

**Figure 3:**
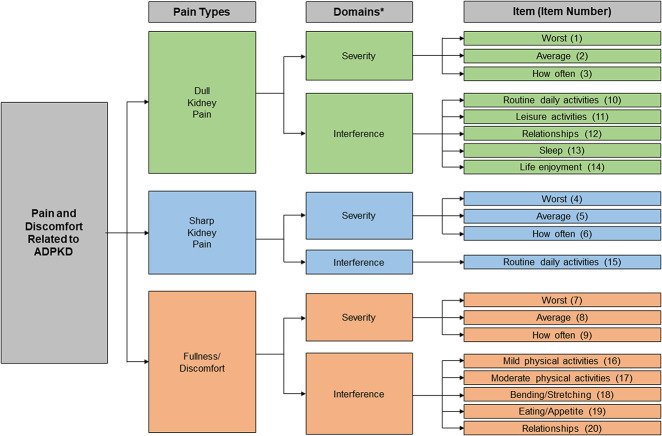
**Final ADPKD-PDS conceptual framework**. Relationships refers to effects on relationships with other people, such as limiting social activities and not spending time with other people. Activities, defined by patients and confirmed in debriefing interviews, consist of leisure activities (such as gardening, hobbies, traveling, playing with children) affected by dull kidney pain, routine daily activities (including walking, bending, light lifting, and housework) affected by dull kidney pain or sharp kidney pain, and mild physical activities (walking, lifting/carrying light objects) and moderate physical activities (brisk walking, dancing, walking upstairs) affected by fullness/discomfort. *In addition, the overall Pain and Discomfort Severity domain is on the basis of the Dull Pain Severity, Sharp Pain Severity, and Discomfort Severity scores.

Our first measurement model made distinctions among the three types of pain (dull, sharp, and discomfort) but combined items asking about the severity of pain events (average event, worst event, and frequency of events) with items asking about the interference of these events on their lives. The comparative fit index and non-normed fit index statistics associated with this model indicated deficiencies that were primarily attributable to correlations among Pain Interference items that should have been distinct. To overcome these structural problems, we developed three major iterations of the ADPKD-PDS measurement model with modifications that included dropping two items related to moodiness, including one addressing the effect of dull pain on moodiness and one addressing the effect of discomfort on moodiness. The final iteration was divided by severity and interference items while retaining the distinction between pain types within each model. The comparative fit and non-normed fit indices were 0.99 for both the pain severity and pain interference models, indicating very good fit (Table 3).^26,27^ The root mean square error of approximation and the standardized root mean square residual were both well within acceptable ranges (<0.06 and <0.05, respectively).^28^ Figure 3 shows the resultant final conceptual framework.

**Table 3:** Autosomal Dominant Polycystic Kidney Disease Pain and Discomfort Scale final model fit statistics

Conceptual Model	χ^2^	df (*P* Value)	CFI	NNFI	RMSEA (90% CI)	SRMR	WRMR
Model 1: Pain severity (*N*=298)	31.7	21 (*P*=0.06)	0.99	0.99	0.041 (0.000 to 0.069)	0.024	0.62
Model 2: Pain interference (*N*=298)	40.7	31 (*P*=0.11)	0.99	0.99	0.032 (0.000 to 0.057)	0.015	0.30

χ^2^, chi-square statistic; df, degrees of freedom; CFI, comparative fit index; NNFI, non-normed fit index; RMSEA, root mean square error of approximation; 90% CI, 90% confidence interval; SRMR, standardized root mean square residual; WRMR, weighted root mean squared residual.

### Clinically Meaningful Change

Clinically meaningful changes in the ADPKD-PDS were explored using both anchor- and distribution-based analyses. Triangulation of results suggests the following thresholds to guide a responder definition: Overall Pain Severity=0.2, Dull Pain Severity=0.5, Sharp Pain Severity=0.5, Discomfort Severity=0.5, Dull Pain Interference=0.2, Sharp Pain Interference=1.0, Discomfort Interference=0.2.

### Clinical Evaluation

Survey participants had a mean age of 48 years (SD 13) and 80% were female (Table 2). Only 13% (*n*=38) had useable total kidney volume information. Nine participants did not report baseline CKD stage and were excluded from the health-related quality of life analysis (*n*=289).

**Figure 4:**
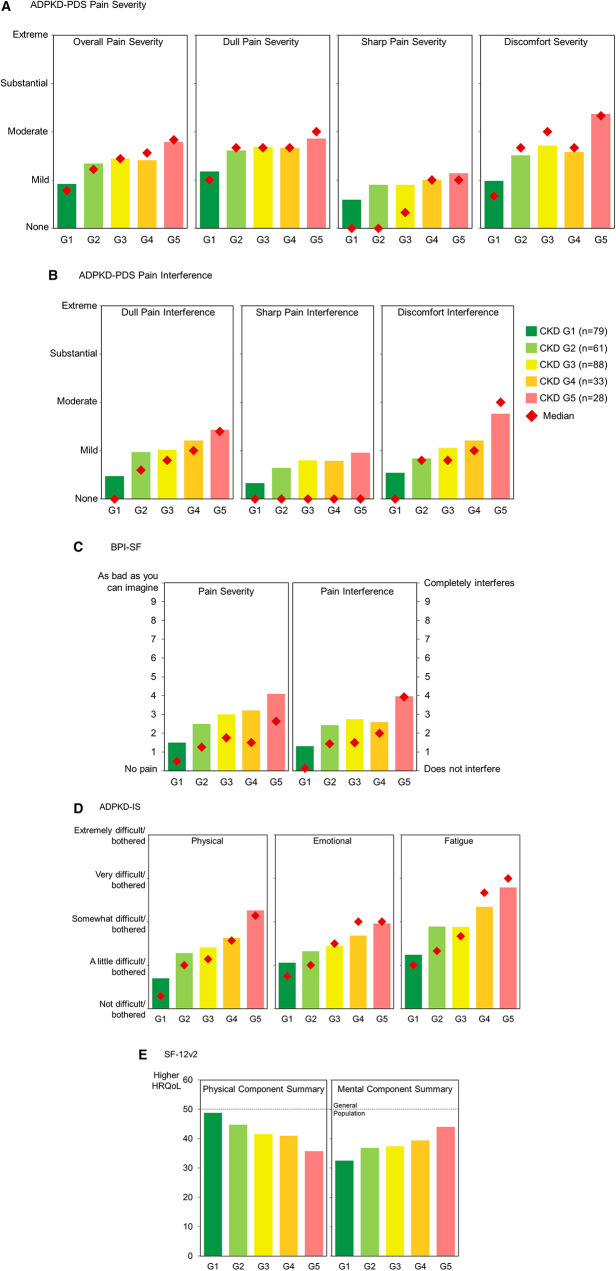
**Mean patient-reported outcomes scores at baseline by CKD stage (*n*=289)**. (A) ADPKD-PDS Pain Severity; (B) ADPKD-PDS Pain Interference; (C) BPI-SF; (D) ADPKD-IS; and (E) SF-12v2. Stage G1: eGFR ≥90 ml/min per 1.73 m^2^; Stage G2: eGFR ≥60 to <90 ml/min per 1.73 m^2^; Stage G3: eGFR ≥30 to <60 ml/min per 1.73 m^2^; Stage G4: eGFR ≥15 to <30 ml/min per 1.73 m^2^; Stage G5: eGFR <15 ml/min per 1.73 m^2^. ADPKD-IS, ADPKD Impact Scale; BPI-SF, Brief Pain Inventory Short Form; HRQoL, health-related quality of life; SF-12v2, 12-Item Short-Form Health Survey version 2.

Results showed that patients in early CKD stages already experience general disease and pain burden, with worse burden at later-stage CKD, following clinically expected patterns (Figure 4).

The ADPKD-PDS showed greater overall pain severity in individuals with more advanced CKD (Figure 4A). Subjective assessment of ADPKD-PDS scores indicated that Dull Pain Severity differentiated least in CKD 2–4 and Sharp Pain Severity was lowest overall, with little differentiation between disease stages. Discomfort Severity was not clearly aligned with CKD stages. Mean and median values for Dull Pain Interference aligned with worse CKD (Figure 4B). Sharp Pain Interference differentiated slightly between CKD stages, and Discomfort Interference showed the most differentiation between CKD stages and the most difference in median values. High variability, presumably due to the rarity of sharp pain events, together with a short recall period of the ADPKD-PDS, likely contributed to lower-than-expected sharp pain scores.

BPI-SF showed greater mean pain severity at later CKD stages, similar to ADPKD-PDS Overall Pain and Discomfort Severity scores, yet with higher variability (Figure 4C). Pain Interference showed more variability and less alignment with CKD stage than ADPKD-PDS Interference scores.

ADPKD-IS scores for all domains showed worse disease burden at later CKD stages (Figure 4D). Patients reported being most affected by fatigue overall and least affected in the physical domain for CKD 1.

SF-12v2 scores showed worse health-related quality of life on the Physical Component Summary and better health-related quality of life on the Mental Component Summary in later-stage CKD (Figure 4E). Overall, patients had lower health-related quality of life than the general US population.

### Final Instrument

The final ADPKD-PDS, resulting from the conceptual framework and confirmatory factor analysis, contains 20 items in seven domains: four Pain Severity domains (Dull Pain Severity, Sharp Pain Severity, Discomfort Severity, and Overall Pain and Discomfort Severity) and three Pain Interference domains (Dull Pain Interference, Sharp Pain Interference, and Discomfort Interference; Figure 3). Access to the full US-English ADPKD-PDS questionnaire and other language versions is available *via* Mapi Research Trust at https://eprovide.mapi-trust.org/.

## Discussion

The ADPKD-PDS was developed using a patient-centered concept elicitation process that included patient and expert clinician input.^13–15^ The instrument was then rigorously evaluated through psychometric analysis. Qualitative research identified that ADPKD-associated pain consists of three distinct pain types not captured by existing PROs: chronic dull kidney pain, chronic discomfort/fullness, and rare/intermittent sharp kidney pain. This is consistent with the finding of the SONG-PKD research group and other investigators that core outcomes in ADPKD, including pain, differ from the experience of other CKDs.^8,16^

Measurement properties of the instrument were well within currently accepted standards for the reliability and validity of PRO assessments. The non-normal distribution of responses to all items was as expected (*i.e.*, not all participants experience all symptoms, and most only experience a particular pain symptom or burden to a small degree). Variations in the percentages of floor responses by item likely reflected the range of ADPKD stages in the sample and the relative likelihood of measured events occurring in the 1-week recall period.

The final ADPKD-PDS is structured on the basis of two broad concepts: pain severity and pain interference. By capturing pain interference with daily life, the instrument addresses not only the core ADPKD outcome of pain but also other outcomes of interest identified by SONG-PKD beyond the core set, such as impacts on the ability to perform everyday physical activities, interact with family and friends, feel rested, and enjoy life.^8^ Another advantage is that by eliciting pain types specific to ADPKD and the ways in which that pain interferes with life activities, the ADPKD-PDS prompts reflection in a population with a tendency to normalize and under-report chronic pain symptoms to which they have become accustomed.^8^ Through use of the instrument, clinical care teams can be made aware of the patient's pain experience that might otherwise remain undisclosed.

The psychometric measurement sample was approximately 80% female, whereas an even distribution between sexes would have been ideal. However, the analysis revealed no substantive differences between sexes for item-level psychometrics; therefore, the large proportion of females should not alter the interpretation of the results. In general, the potential for sampling bias is a drawback of convenience sampling and must be acknowledged as a limitation. Although the focus groups were convened internationally, recruitment for the online survey was conducted only in the United States.

Another limitation is the short 7-day recall period of the ADPKD-PDS, which, combined with the relatively short study period, may have reduced the chance of capturing episodic or sporadic sharp pain symptoms. The question of the most appropriate recall period points to the potential for tracking apps and real-time reporting technology to improve PRO collection. Research conducted using the ADPKD-PDS might be enhanced, for example, by employing experience sampling methods^29^ in which respondents are asked a brief series of additional questions to capture their moods, thoughts, and symptoms at the time of ADPKD-PDS completion. This approach can reduce the effects of recall bias and capture real-life context leading to responses on the ADPKD-PDS. Finally, due to the relative rarity of sharp kidney pain in the studied population, there was an inadequate structural basis for a multi-item Sharp Pain Interference domain, and a single item assesses this outcome. With use of the ADPKD-PDS in larger studies of longer duration, the infrequency of sharp kidney pain might be less of an issue. Implementation of the ADPKD-PDS as an outcome measure in clinical studies was planned for soon after the instrument was developed, but the authors do not have these data available.

Finally, the use of pain medications may also have influenced the interpretation of the data; 28% of participants reported taking pain medication, but additional information on pain treatment beyond this yes/no question was not collected.

The survey results showed a greater pain burden on the ADPKD-PDS and worse health-related quality of life on the SF-12v2 Physical Component Summary at more advanced ADPKD stages, whereas the SF-12v2 Mental Component Summary indicated better mental health at later CKD stages. This finding is to be expected, as mental health may paradoxically improve as individuals age and their health worsens.^30^ After ADPKD diagnosis, patients may also gradually become more accepting of their condition. Recruitment from a patient advocacy organization may have skewed the sample toward individuals more educated or motivated about their disease.

The ADPKD-PDS provides the first dedicated tool for systematically assessing pain and discomfort burden across CKD stages in ADPKD. Given the relatively few pain symptoms at baseline in early-stage ADPKD, the use of the ADPKD-PDS in interventional trials conducted in such subpopulations would require an adequate duration of follow-up to show a treatment effect over time. The psychometric evaluation demonstrated that the instrument is responsive to change over time and is therefore suitable to capture longitudinal changes in pain burden in patients with ADPKD. As the ADPKD-PDS was developed in an adult population, a pediatric pain assessment instrument in ADPKD is still needed, given that a substantial proportion of such patients report such symptoms.^5^ Nonetheless, concepts reported by adult patients in the qualitative research for development of the ADPKD-PDS are supported by pediatric/adolescent qualitative research.^5^

In previous research, individuals with ADPKD identified inadequate pain management and trivialization of their pain by physicians as key concerns, pointing to the need for tools to address ADPKD-related pain.^31^ The rigorous development and testing of the ADPKD-PDS have resulted in a tool that meets or exceeds accepted standards for reliability and validity of PRO instruments. Moreover, the involvement of focus groups across a wide variety of geographical and cultural regions showed consistent findings, suggesting that the concepts assessed by the instrument are generalizable to the global ADPKD population. Given the growing interest in patient-centered research, development of the ADPKD-PDS may lead to a better understanding of how to improve the symptomatic effect of pain in people living with ADPKD.

## Supplementary Material

## Supplemental Material

Literature Search Methodology.

Supplemental Table 1. Literature search terms.

Focus Group Methodology.

Results of Quantitative Analyses.

Supplemental Table 2. Item-level characteristics of the ADPKD-PDS (*N*=298).

Supplemental Table 3. Distributional and internal consistency statistics of ADPKD-PDS domains (*N*=298).

Supplemental Table 4. Correlations of the ADPKD-PDS with other instruments at baseline (*N*=298).
