# Supplementary material for: Development of a Patient-Reported Outcomes Tool to Assess Pain and Discomfort in Autosomal Dominant Polycystic Kidney Disease
Source: Clin J Am Soc Nephrol. 2023 Jan 2;18(2):213–22. doi: 10.2215/CJN.0000000000000034 (PMC10103266; doi:10.2215/CJN.0000000000000034)
Supplement: Supplementary file 1 [file cjasn-18-213-s001.pdf]

SUPPLEMENTAL MATERIAL

Contents

Literature Search Methodology

Supplemental Table 1. Literature search terms Focus Group Methodology Results of Quantitative Analyses

Supplemental Table 2. Item-level characteristics of the ADPKD-PDS (N=298)

Supplemental Table 3. Distributional and internal consistency statistics of ADPKD-PDS domains (N=298)

Supplemental Table 4. Correlations of the ADPKD-PDS with other instruments at baseline (N=298)

References

Literature Search Methodology

A literature search was conducted in 2010 to identify articles pertaining to health-related quality of life and other patient-reported outcomes (PROs) used in autosomal dominant polycystic kidney disease (ADPKD) studies. Medical Literature Analysis and Retrieval System Online (MEDLINE®) was used for the initial literature review. A list of the search terms that were used is provided in **Supplemental Table 1**.

A supplementary literature search was conducted in September 2014 to confirm findings and included the term "ADPKD pain." The literature search was limited to papers published in 2004-2014.

Supplemental Table 1. Literature search terms

	MeSH Terms	EMTREE Terms	Title/Abstract Terms
Disease	• Polycystic kidney diseases<br>• Polycystic kidney, autosomal dominant	• Kidney disease<br>• Autosomal dominant inheritance	
	And		
PRO	• Nocturia<br>• Sleep<br>• Anxiety<br>• Depression<br>• Quality of life<br>• Guilt<br>• Personal satisfaction<br>• Patient satisfaction<br>• Mental health<br>• Health status<br>• Thirst<br>• Polyuria	• Nocturia<br>• Sleep<br>• Anxiety<br>• Depression<br>• Quality of life<br>• HRQoL<br>• Guilt<br>• Mental health<br>• Satisfaction<br>• Health status<br>• Burden<br>• Micturition disorder	• Patient reported<br>• Patient-reported<br>• Instruments<br>• Functional status<br>• HRQoL<br>• Health utility<br>• Preference<br>• Quality of life<br>• Well-being<br>• Global impression<br>• Productivity

HRQoL, health-related quality of life; MeSH, medical subject heading; PRO, patient-reported outcome.

Focus Group Methodology

Discussion guides were used to facilitate the focus groups. All study documents were translated into the appropriate languages by in-country specialized medical translators, all of whom had noted experience with renal disease translations. Focus groups in English-speaking locations were moderated by English speakers (the study research scientist and study investigator, respectively). Focus groups conducted in non-English speaking locations used trained moderators to facilitate the discussions in native languages. These focus group discussions were monitored by the study investigator using simultaneous translation in the adjoining back room. Study investigators observed all focus group interactions, including before, during, and after the formal meeting took place. When a translator was being used, translation continued during these times to allow for such observations to be replicated in all sites.

Focus group recordings were transcribed verbatim by a professional vendor. For focus groups conducted in a non-English-speaking country, transcription was simultaneously conducted from the English interpretation audio file. During data transcription, participants were referred to by number only. If a participant was identified on the recording, his or her identity was changed to a number at the time of transcription. No data were linked to any particular participant.

Transcripts of focus group sessions were reviewed and codified for analyses. The data analyses conducted were primarily descriptive (e.g., means, standard deviations). Concepts were identified based on themes that were mentioned by 2 or more participants within a group. Saturation, the point at which no new concepts are identified, was considered to be achieved when no new concepts or themes were identified during subsequent focus groups or interviews. A saturation table was developed based on the participants’ responses from the focus groups.

Results of Quantitative Analyses

Item response theory

The item response theory analysis examined the ability of each item to distinguish among persons with different levels of the trait. Item discrimination generally ranges from 0.00 to 5.00; a larger discrimination parameter indicates an item can make finer distinctions between persons.[1] Using a 2-parameter logistic (2PL) graded-response model, we did not observe any problems with item or scale performance of sufficient concern to warrant dropping or rescaling any item. Item discrimination was strong across Pain Severity items and scales (2.19-3.40) and no items had weak discrimination. The 2PL discrimination parameters of the Pain Interference items (3.09-12.11) were generally higher than those of Pain Severity items. To determine the scoring method, we compared a simple mean score approach with item response theory–based scoring. For the Overall Pain and Discomfort Severity and Total Pain Interference scales, the Pearson correlation coefficients between the two scoring methods were high, 0.97 and 0.95, respectively. As correlations >0.90 indicate method redundancy, there was no reason to use the more complicated item response theory–based scoring approach, and thus the final ADPKD-PDS scoring algorithm uses simple mean scores.[2]

Differential item performance by gender

Differential item function analyses were conducted by gender groups. A series of nested ordinal logistic regression models allowed for detection of uniform (main effects observed for group after accounting for the latent trait) and non-uniform differences in the latent trait observed by gender groups. Uniform differential item function affects high and low scorers on the continuum similarly and is observed as a uniform shift in the regression line that is explained, in this case, by gender. Non-uniform differential item function affects high or low scorer in by gender differently and is

indicative of an interaction effect of, in this case, gender by level of the trait being assessed. Uniform differential item function is considered a more problematic indicator of differential item performance and is often handled by removal of the item from the assessment.[3,4]

No uniform differential item function was observed for any item on the ADPKD-PDS, although non-uniform differential item function was observed for 4 items. Examination of residual regression plots and standardized beta-coefficients for these items revealed few obvious differences. For item 16, males tended to have a higher variation in the reported frequency of sharp pain ratings than females – this weakened the precision in the model, which was compounded by smaller number of males (n=59) than females (n=239) in the study or possible differences in sample characteristics of male versus female participants. Nothing stood out as a reason to drop these items or nor apply a different item scoring algorithm by gender.

Item-level psychometric statistics

The adjusted item-total correlations (correlation between the item score and the domain score) ranged from 0.69 to 0.77 for the Overall Pain Severity items, 0.80 to 0.83 for Dull Pain Severity items, 0.73 to 0.81 for Sharp Pain Severity items, and 0.85 to 0.91 for Discomfort Severity items, all of which exceed the recommended minimum value of 0.40.[5] Moreover, each item had a higher correlation with its own scale than with other scales in the measure, and its relationship with other conceptually related scales was stronger than with less directly related scales. These observations reflect the strong structural characteristics of the Pain Severity and Pain Interference scales. Logistic regression-based item bias analysis showed no substantial difference between genders.

Domain-level descriptive statistics

Internal consistency of the domains was high, indicating good reliability (**Supplemental Table 3**). Cronbach's alpha for each domain ranged from 0.87 to 0.95 and, as one would expect, was higher with a Spearman- Brown correction to a 10-item scale (0.93-0.98). Also as expected, average correlations between items within each Pain Severity scale were generally high (0.71-0.85). Overall Pain Severity was influenced by the heterogeneity of the pain items across the three pain domains and had the lowest inter-item correlation (r_{ii}=0.57). Substantial floor effects (the percentage of participants reporting the lowest score) were observed for all domains (13.1%-70.1%). The highest floor effects were observed for Sharp Pain Severity and Sharp Pain Interference, reflecting the relative rarity of this symptom. Minimal ceiling effects (percentage of participants reporting the highest score) for the domains were observed (0.3%-4.0%).

Convergent validity

Correlations between the ADPKD-PDS Pain Severity scales and the BPI-SF Intensity scale (0.56-0.76) reflected strong convergence, as did correlations between the ADPKD-PDS Pain Interference scales and the BPI-SF Impact scale (0.59-0.84), likely because these scales have very similar measurement constructs (**Supplemental Table 4**). Correlations with the SF-12v2 were lower due to less similar, yet related constructs. The Pain Interference scales correlated more strongly with the SF-12v2 Physical Component Scale and Mental Component Scale (-0.37 to -0.67) than did the Pain Severity scales (-0.35 to -0.58). Correlations were also moderate to high between ADPKD-PDS scales and the ADPKD-IS Physical, Fatigue, and Emotional scales (0.37-0.76 for ADPKD-PDS Pain Severity scales and 0.40-0.86 for ADPKD-PDS Pain Interference scales). Taken together, these results provide evidence that both the ADPKD-PDS Pain Severity and Pain Interference scales possess good convergent validity characteristics.

Longitudinal characteristics

Test-retest reliability. Except for a single item in the Sharp Pain Interference scale, the test-retest correlations for all domains (0.73-0.90) exceeded the commonly accepted threshold of 0.70.[6] The Cohen's d effect sizes for changes in item scores were low (0.01 to 0.12) and similar to those for domain scores (0.01 to 0.11). The distribution of change scores and the small standardized mean differences in domain scores between the first and second administration of the ADPKD-PDS indicate little change in symptoms over time, consistent with the slow disease progression of ADPKD.

Responsiveness to change. Understanding what constitutes meaningful change helps clinicians make treatment decisions. Distribution-based responder analysis of changes in scores using a responder definition based on the standard error of measurement indicated that a change of 0.2-0.5 points (4%-10%) or more in either direction represents meaningful change for six of the seven ADPKD-PDS scales. An exception is the Sharp Pain Interference scale, which requires a change of a full point to be considered clinically meaningful (this is a single-item scale; a change of less than a full point cannot be estimated using a single item). Anchor-based responder analysis, in which other instruments are used as the reference for change, showed that five of the seven ADPKD-PDS scales were responsive to change in the pain-based global rating of change, six scales were responsive to change in the BPI-SF Pain Intensity scale, and all three Pain Interference scales were responsive to change in the BPI-SF Impact scale. This indicates that the ADPKD-PDS captured small but meaningful change over time.

Supplemental Table 2. Item-level characteristics of the ADPKD-PDS (N=298)

Items[a]	Score Range	Item Mean (SD)	Median	% (n) of Floor Responses	% (n) of Ceiling Responses	Skew Z-Score	Normal Pr Skew	Kurtosis Z-Score	Normal Pr Kurtosis
Item 1	1-5	2.71 (1.24)	3	22.2% (66)	7.7% (23)	0.69	0.48	-3.61	0.000
Item 2	1-4[b]	2.12 (0.92)	2	29.2% (87)	7.4% (22)	2.47	0.02	-2.78	0.000
Item 3	1-5	2.78 (1.31)	3	25.5% (76)	7.7% (23)	-0.47	0.63	-4.40	0.000
Item 4	1-5	1.89 (1.06)	1.5	50.0% (149)	0.7% (2)	6.11	0.000	-1.63	0.04
Item 5	1-5	1.98 (1.18)	2	49.0% (146)	3.4% (10)	6.72	0.000	-0.84	0.40
Item 6	1-5	1.68 (1.04)	1	62.8% (187)	2.0% (6)	10.13	0.000	3.93	0.004
Item 7	1-5	2.04 (1.19)	2	46.3% (138)	3.4% (10)	5.86	0.000	-1.65	0.04
Item 8	1-5	1.97 (1.18)	2	49.7% (148)	4.7% (14)	7.19	0.000	0.17	0.77
Item 10	1-5	2.09 (1.47)	1	57.0% (170)	12.4% (37)	6.86	0.000	-2.22	0.002
Item 11	1-5	1.75 (1.07)	1	59.4% (177)	1.3% (4)	8.83	0.000	1.39	0.18
Item 12	1-5	1.67 (0.99)	1	62.1% (185)	0.7% (2)	9.09	0.000	1.88	0.09
Item 13	1-5	1.64 (1.14)	1	70.1% (209)	4.0% (12)	12.02	0.000	5.97	0.0002
Item 14	1-5	2.60 (1.33)	3	30.2% (90)	9.1% (27)	1.61	0.11	-4.14	0.0000
Item 15	1-5	2.22 (1.13)	2	33.9% (101)	2.7% (8)	3.95	0.0002	-2.35	0.0005
Item 16	1-5	2.64 (1.34)	3	30.5% (91)	7.7% (23)	0.74	0.45	-4.72	0.0000
Item 17	1-5	1.84 (1.07)	1	54.7% (163)	1.3% (4)	6.96	0.000	-0.62	0.57
Item 18	1-5	1.92 (1.16)	1	52.0% (155)	3.0% (9)	7.22	0.000	-0.37	0.79
Item 19	1-5	2.08 (1.22)	2	45.3% (135)	4.0% (12)	5.67	0.000	-1.89	0.013
Item 20	1-5	2.14 (1.19)	2	42.0% (125)	3.0% (9)	4.50	0.000	-2.74	0.000
Item 21	1-5	1.70 (1.03)	1	60.4% (180)	1.7% (5)	9.81	0.000	3.54	0.008

ADPKD-PDS, Autosomal Dominant Polycystic Kidney Disease–Pain and Discomfort Scale; CFA, confirmatory factor analysis; Pr, probability; SD, standard deviation.

[a] Items 9 and 22 were removed from the Pain Interference scales during CFA modeling.

[b] No respondents endorsed the Extreme rating for their average dull pain.

Supplemental Table 3. Distributional and internal consistency statistics of ADPKD-PDS domains (N=298)

Domain	Item Mean (SD)	Observed Range	% Floor[a]	% Ceiling[b]	Mean r_{ii}	Cronbach's Alpha	Spearman-Brown Adjusted Alpha (k=10)[c]
Dull Pain Severity (k=3)	2.54 (1.06)	(1.00-4.67)	19.1%	0.7%	0.76	0.90	0.97
Sharp Pain Severity (k=3)	1.84 (1.06)	(1.00- 4.67)	52.0%	0.7%	0.71	0.87	0.96
Discomfort Severity (k=3)	2.49 (1.20)	(1.00-5.00)	28.5%	1.7%	0.85	0.94	0.98
Overall Pain/Discomfort Severity (k=9)	2.29 (0.94)	(1.00-4.56)	13.1%	0.3%	0.57	0.92	0.93
Dull Pain Interference (k=5)	1.91 (1.03)	(1.00-5.00)	36.6%	0.7%	0.80	0.95	0.97
Sharp Pain Interference (k=1)	1.64 (1.14)	(1.00-5.00)	70.1%	4.0%	--	--	--
Discomfort Interference (k=5)	1.94 (1.03)	(1.00-4.80)	35.6%	0.3%	0.79	0.95	0.97

k, number of items; r_{ii}, inter-item correlation; SD, standard deviation.
[a] Percentage of patients with lowest observed score in the range
[b] Percentage of patients with highest observed score in the range
[c] Cronbach's alpha with a Spearman-Brown correction to a 10-item scale

Supplemental Table 4. Correlations of the ADPKD-PDS with other instruments at baseline (N=298)

	ADPKD-PDS Scales						
**Instrument Scales**	**Overall Pain Severity**	**Dull Pain Severity**	**Sharp Pain Severity**	**Discomfort Severity**	**Dull Pain Interference**	**Sharp Pain Interference**	**Discomfort Interference**
**ADPKD-PDS**							
Dull Pain Severity	0.86						
Sharp Pain Severity	0.84	0.63					
Discomfort Severity	0.85	0.59	0.53				
Dull Pain Interference	0.78	0.77	0.60	0.63			
Sharp Pain Interference	0.73	0.57	0.81	0.49	0.64		
Discomfort Interference	0.77	0.56	0.55	0.82	0.74	0.58	
**BPI-SF**							
Intensity	0.76	0.76	0.62	0.56	0.75	0.61	0.59
Impact	0.70	0.67	0.56	0.56	0.84	0.59	0.71
**SF-12v2**							
Physical Component Scale	-0.57	-0.58	-0.40	-0.48	-0.67	-0.44	-0.61
Mental Component Scale	-0.43	-0.40	-0.35	-0.35	-0.52	-0.37	-0.46
**ADPKD-IS**							
Physical Function	0.76	0.71	0.57	0.65	0.86	0.61	0.79
Fatigue	0.66	0.58	0.52	0.57	0.70	0.53	0.68
Emotional Distress	0.52	0.42	0.37	0.52	0.61	0.40	0.61

ADPKD-IS, Autosomal Dominant Polycystic Kidney Disease–Impact Scale; ADPKD-PDS, Autosomal Dominant Polycystic Kidney Disease–Pain and Discomfort Scale; BPI-SF, Brief Pain Inventory–Short Form; SF-12v2, Medical Outcomes Study 12-Item Short-Form Health Survey version 2.

All table values are Pearson product-moment correlation coefficients (Pearson's r).

Shading key:

>0.80

0.60 – 0.79

0.50 – 0.59

0.30 – 0.49

<0.30

References

1. Samejima F: Graded response model. In: van der Linden WJ, Hambleton RK, eds. Handbook of Modern Item Response Theory. 85–100. New York, NY: Springer, 1997
2. Ware JE, Harris W, Gandek B, Rogers B, Reese P: MAP-R for Windows: Multitrait/Multi-Item Analysis Program—Revised User's Guide. Boston, MA: Health Assessment Lab, 1997
3. Glas CAW, Verhelst ND: Tests of fit for polytomous Rasch models. In: Fischer GH, Molenaar IW, eds. Rasch Models—Foundations, Recent Developments, and Applications. 325–352. Berlin, Germany: Springer, 1995
4. Crane PK, Gibbons LE, Jolley L, van Belle G: Differential item functioning analysis with ordinal logistic regression techniques. DIFdetect and difwithpar. Med Care 44 (11 Suppl 3): S115–23, 2006
5. Martin BC, Pathak DS, Sharfman MI, Adelman JU, Taylor F, Kwong WJ, Jhingran P: Validity and reliability of the migraine-specific quality of life questionnaire (MSQ Version 2.1). Headache 40: 204-15, 2000
6. Terwee CB, Bot SD, de Boer MR, van der Windt DA, Knol DL, Dekker J, Bouter LM, de Vet HC: Quality criteria were proposed for measurement properties of health status questionnaires. J Clin Epidemiol 60: 34-42, 2007
